# Evaluation of the Extraction Temperature Influence on Polyphenolic Profiles of Vine-Canes (*Vitis vinifera*) Subcritical Water Extracts

**DOI:** 10.3390/foods9070872

**Published:** 2020-07-03

**Authors:** Olena Dorosh, Manuela M. Moreira, Diana Pinto, Andreia F. Peixoto, Cristina Freire, Paulo Costa, Francisca Rodrigues, Cristina Delerue-Matos

**Affiliations:** 1 REQUIMTE/LAQV, Instituto Superior de Engenharia do Instituto Politécnico do Porto, Rua Dr. António Bernardino de Almeida, 431, 4249-015 Porto, Portugal; olena.dorosh@graq.isep.ipp.pt (O.D.); manuela.moreira@graq.isep.ipp.pt (M.M.M.); diana.pinto@graq.isep.ipp.pt (D.P.); cmm@isep.ipp.pt (C.D.-M.); 2 REQUIMTE/LAQV, Departamento. de Química e Bioquímica, Faculdade de Ciências, Universidade do Porto, Rua do Campo Alegre s/n, 4169-007 Porto, Portugal; andreia.peixoto@fc.up.pt (A.F.P.); acfreire@fc.up.pt (C.F.); 3 REQUIMTE/UCIBIO, MedTech-Laboratory of Pharmaceutical Technology, Department of Drug Sciences, Faculty of Pharmacy, University of Porto, Rua de Jorge Viterbo Ferreira nº. 228, 4050-313 Porto, Portugal; pccosta@ff.up.pt

**Keywords:** vine-canes, subcritical water extraction, scavenging capacity, in-vitro assays, byproducts

## Abstract

This work focused on evaluating the possibility of using vineyard pruning wastes from two Portuguese *Vitis vinifera* varieties; Touriga Nacional (TN) and Tinta Roriz (TR), as new potential ingredients for the nutraceutical industry. An environmentally friendly extraction technique; namely subcritical-water extraction (SWE), was employed. The overall results indicate that phenolic acids were the major class of compounds quantified; being gallic acid the principal one. The highest value for total phenolic content (TPC) was obtained for the TR extract at 250 °C (181 ± 12 mg GAE/g dw). In terms of antioxidant activity; the DPPH values for the extracts obtained at 250 °C were approximately 4-fold higher than the ones obtained at 125 °C; with TR extract presenting the highest value (203 ± 22 mg TE/g dw). Thus, the TR extract obtained through SWE at 250 °C was selected to evaluate the scavenging activity and the in vitro effects on cells due to the best results achieved in the previous assays. This extract presented the ability to scavenge reactive oxygen species (O_2_^●-^, HOCl and ROO^●^). No adverse effects were observed in HFF-1 viability after exposure to extract concentrations below 100 μg/mL. This work demonstrated that vine-canes extracts could be a potential ingredient to nutraceutical industry

## 1. Introduction

Contemporary society is tightly bonded to over-consumerism being characterized by a mass-production of goods and consequently their over consumption. This linear economy depends on two basic assumptions: (i) there will always be resources that can be extracted and (ii) there will always be a place where it is possible to get rid of the materials that are not wanted anymore. Nevertheless, currently, due to the development of science and environmental awareness, there is a growing conscience that these two assumptions are not real and that the linear economy is not sustainable [1]. In fact, human population has grown exponentially in the last two centuries increasing the resources consumption [2]. The increased demand over natural resources has been negatively affecting Earth’s overshoot day. To reach a more sustainable world status and to preserve what is still left for future generations, it is imperative the transition from an economy based on fossil resources to a concept of circular economy. A growing number of companies, including the food industry, are working to overcome this challenge and transform their process of production in a more environmentally ethical practice. Food and beverage industries are the principal manufacturing sector in the European Union (EU) generating high amounts of byproducts for which profitable solutions need to be found [3,4]. Until now, the general application for the agro-food industry waste has been animal feed (that may not adjust to the nutritional requirements), combustion feedstock or fertilizers, causing major environmental issues [5,6]. Nevertheless, these byproducts can be used as renewable natural resources for many applications, such as low-cost adsorbents, nutraceuticals, supplement food products and ready meals, leading these industries to a concept more related to a circular economy [6,7].

Grapes (*Vitis vinifera*) are one of the principal fruits produced around the world [8]. According to OIV (International Organization of Vine and Wine), in the last years, Portugal is one of the principal world wine producers, representing this sector as a huge impact on the economy [9]. Consequently, large amounts of wine wastes, namely skins, seeds and stems are produced every year, representing approximately 20% of the total weight of processed grapes [10]. These undervalued byproducts are rich in bioactive compounds, particularly polyphenols [11,12,13,14,15], that could be used in several applications such as antioxidants in food, cosmetic or even pharmaceutical industries [16]. Depending on the vine varieties, around 1.75 tons of vine-cane wastes are produced for each hectare of vineyard. After harvesting season, many agricultural byproducts, including vine-canes, are usually incorporated in the soil, enhancing the soil health due to the degradation of organic matter and reducing the necessities of organic fertilizers and/or correctives [17]. Following the need to find a better end for vine-canes, different approaches were already explored, such as the biochar production, biofuels, pulp for paper sheets and particle board [18,19,20].

Vine-canes are constituted by two main fractions, holocellulose (cellulose and hemicellulose) and lignin, which correspond to approximately 68% and 20% of the total vine-canes weight, respectively [21]. The lignin degradation process releases phenolic compounds of low molecular weight, alcohols, aldehydes, ketones or acids [16]. By employing the appropriate extraction technique, considerable amounts of these valuable compounds can be recovered from the lignin fraction of vine-canes and afterwards used in added valued products. Subcritical water extraction (SWE) can be exploited as a sustainable and clean technique to achieve this goal. SWE is a pressurized liquid extraction technique that uses water at high temperatures (100–374 °C) and pressures (1–22.1 MPa), but below its critical point (374 °C and 22.1 MPa). The use of water as extracting solvent makes this technique safe, cost-effective and environmentally interesting, particularly for the extraction of phenolic compounds to be used in products for human consumption. For example, Gabaston et al. investigated the effect of different times (5, 15 and 30 min) and temperatures (100, 130, 160 and 190 °C) in SWE [22]. All extractions were conducted with 5 g of vine-canes powder in a 34 mL cartridge. According to the authors, the best results were obtained at 160 °C for 5 min (3.62 g of stilbenes/kg dry weight (dw)) [22]. Indeed, in our previous study, we compared three extraction techniques, namely microwave-assisted extraction (MAE), SWE and conventional extraction (CE), in what concerns to phenolic compounds extracted from vine-canes [12]. The obtained results revealed the advantages and potentialities of SWE, when compared to the other techniques. In this study, we focused on the extraction of vine-canes from two Portuguese varieties (Touriga Nacional, TN and Tinta Roriz, TR) from the Dão region using SWE performed at two different temperatures (125 and 250 °C) in order to maximize the bioactive compounds extraction. Opposite to our previous study, where we establish a specific temperature for the SWE (150 °C) in this work we aim to compare different extraction temperatures taking in consideration the results reported by other authors highlight temperature as the most influencing parameter on SWE [23,24,25]. Thus, based on the previous studies, we decided to explore the thermo-chemical conversion reactions effects on the recovery of antioxidant rich products using subcritical water conditions. Based on this, the antioxidant activities and the phenolic profile of the two vine-canes varieties were investigated. The best extract was selected for further assays, being screened the scavenging activity against oxygen radical species as well as the cell viability effects. Overall, this work follows a sustainable approach for the valorization of vine-canes from the grape industry in order to promote their added value and circular economy.

## 2. Materials and Methods

### 2.1. Samples Collection and Preparation

Vine-canes samples were kindly provided by Sogrape Vinhos, S.A. (Portugal). Both *V. vinifera* vine-canes varieties, namely TN and TR, were obtained in Quinta dos Carvalhais, located in Mangualde (North of Portugal), in November 2015 by randomized selection. Samples were oven-dried (Model no. 2000208, J.P. Selecta, Barcelona, Spain) at 50 °C for 24 h and milled (Retsch ZM200) to a particle size smaller than 1 mm. The fine particles obtained after milling were stored in sealed bags at room temperature until use.

### 2.2. Subcritical Water Extraction

**Figure 1:**
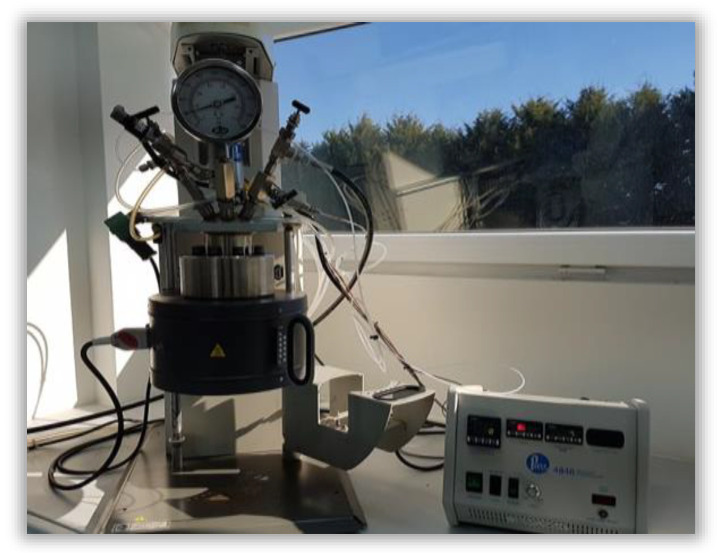
Subcritical water extractor used.

SWE was conducted in a Parr Series 4560 Reactor connected to the Parr 4848 Reactor Controller (Figure 1).

The extractions were performed using 40 g of milled vine-canes and 400 mL of water. Two different extraction temperatures were tested, 125 and 250 °C, for 50 min after the sample reached the desired temperature at 250 rpm. After extraction, the system was cooled down and the extract was filtered and centrifuged at 15,763× *g* (Heraeus Megafuge 16 Centrifuge Series, Thermo Scientific, Waltham, MA, USA) for 15 min at 4 °C. Then, the extract was stored at −80 °C and lyophilized (Edwards lyophilizer) for 48 h, after being stored at 4 °C until further use.

### 2.3. Determination of Total Phenolic and Flavonoid Contents

The total phenolic content (TPC) was determined according to Singleton and Rossi [26], with minor modifications described by Paz et al. [27]. The reaction solution consisted of 25 µL of deionized water (blank), standard or sample, 75 µL of deionized water and 25 µL of diluted Folin–Ciocalteu reagent (1:1). After 6 minutes in the dark, 100 µL of a sodium carbonate solution (0.708 M) were added to each well. The microplate (BioTek Instruments, Inc., Winooski, VT, USA) was kept away from the light for 90 min and then the absorbance was measured at 760 nm. Calibration curves were done using gallic acid (GA) as standard. Results were expressed as milligrams of gallic acid equivalents (GAE) per gram of dw (mg GAE/g dw).

The total flavonoid content (TFC) was performed according to Paz et al. [27]. The procedure consisted in adding to each well 100 µL of deionized water, 10 µL of sodium nitrite solution (0.725 M) and 25 µL of deionized water (blank) or standard or sample. After 5 min in the dark, 15 µL of aluminum chloride (0.75 M) were added to each well, and after 1 minute of reaction, also in the dark, 50 µL of sodium hydroxide (1.0 M) were added. The absorbance was measured at 510 nm. Epicatechin was used as standard. Results were expressed as mg of epicatechin equivalents (EE) per gram of dw (mg EE/g dw).

### 2.4. Determination of Antioxidant Activity and DPPH Free Radical Scavenging Assay

The FRAP assay was based on the protocol reported by Benzie and Strain [28], with minor modifications described by Paz et al. [27]. FRAP reagent was prepared using a mixture of acetate buffer (pH 3.6; 0.3 M), 2,4,6-Tri(2-pyridyl)-s-triazine (TPTZ; 0.01 M) in HCl solution (0.04 M) and FeCl_3_.6H_2_O (0.27 M) in a 10:1:1 ratio. One hundred and eighty microliters of FRAP reagent were added to each well of the microplate along with 20 µL of deionized water (blank) or standard or sample. Absorbance was measured at 593 nm, after incubating in the dark at 37 °C for 10 min. A calibration curve was prepared with ascorbic acid (AA). Results were expressed as milligrams of AA equivalents (AAE) per gram of dw (mg AAE/g dw).

DPPH-RSA was performed following the protocol described by Paz et al. [27]. For that, 200 µL of an ethanolic solution of DPPH (0.1 M) were added to 25 µL of the standard or sample. The blank contained 225 µL of ethanol and the control 225 µL of the DPPH reagent. The reaction solution was incubated for 30 min in the dark. DPPH-RSA was determined spectrophotometrically at 517 nm. Calibration curve was made with Trolox. Results were expressed in milligrams of Trolox equivalents (TE) per gram of dw (mg TE/g dw).

### 2.5. Qualitative and Quantitative Polyphenol Characterization

The phenolic profile of subcritical water extracts was obtained by high performance liquid chromatography (HPLC) with photodiode array (PDA) detection employing the method described by Moreira et al. [12]. A Shimadzu HPLC system equipped with a Phenomenex Gemini C_18_ column (250 mm × 4.6 mm, 5 μm) and a guard column with the same characteristics, that were kept at 25 °C, were used. Individual phenolic compounds were prepared in methanol and their mixtures for calibration curves construction were obtained by dilution of appropriate amounts of the stock solutions in methanol:water 50:50 (*v*/*v*). The mobile phase was composed by methanol (A) and water (B) both with 0.1% formic acid, which were previously filtered (0.20 μm nylon filter, Supelco, Bellefonte, PA, USA) and degassed for 15 min in an ultrasonic bath (Raypa^®^ trade, Terrassa, Spain). A gradient program, at a flow rate of 1.0 mL/min and 20 μL of injection volume were used and the identification of detected peaks in subcritical water extracts was performed by comparing their retention time and UV-vis spectra with the ones of pure standards. GA, protocatechuic acid, (+)-catechin, 4-hydroxyphenilacetic acid, 4-hydroxybenzoic acid, 4-hydroxybenzaldehyde, vanillic acid, syringic acid, (-)-epicatechin, naringin, phloridzin, cinnamic acid, naringenin, phloretin and pinocembrin were quantified at 280 nm; chlorogenic acid, caffeic acid, *p*-coumaric acid, ferulic acid, sinapic acid, resveratrol and tiliroside at 320 nm and quercetin-3-*O*-glucopyranoside, rutin, ellagic acid, myricetin, kaempferol-3-*O*-glucoside, kaempferol-3-*O*-rutinoside, quercetin and kaempferol at 360 nm and their amount was expressed as mg/100 g dw.

### 2.6. Reactive Oxygen Species Scavenging Capacity

#### 2.6.1. Superoxide Radical Scavenging Assay

The superoxide radical (O_2_^●-^) scavenging assay was performed according to Pistón et al. [29]. The reaction mixture was prepared by adding to each well the following reagents dissolved in phosphate buffer (19 × 10^−3^ M, pH 7.4): 50 µL of NADH (166 × 10^−6^ M); 150 µL of nitroblue tetrazolium (NBT; 43 × 10^−6^ M); 50 µL of tested extract at different concentrations or phosphate buffer for the blank or positive controls and finally 50 µL of PMS (2.7 × 10^−6^ M). Absorbance was read at 560 nm for 6 min at 37 °C in the microplate reader. GA and catechin were used as positive controls. The observed effects were expressed as inhibition percentages of the NBT reduction to diformazan. Results were expressed as the necessary concentration of controls and subcritical water extract of TR variety obtained at 250 °C to inhibit 50%, IC_50_, of the NBT reduction to diformazan.

#### 2.6.2. Hypochlorous Acid Scavenging Activity

Hypochlorous acid (HOCl) scavenging activity was measured using a fluorescent methodology previously described by Pistón et al., based on the HOCl-induced oxidation of dihydrorhodamine (DHR) to rhodamine [29]. GA and catechin were used as positive controls. A 1% (*m*/*v*) NaOCl solution was used to prepare the HOCl solution by adjusting the pH to 6.2 with addition of H_2_SO_4_. The assay was directly performed in a 96-well microplate and the reagents previously dissolved in phosphate buffer (100 × 10^−3^ M, pH 7.4). In each well, the reaction mixture consisted of: 150 µL of phosphate buffer (100 × 10^−3^ M); 50 µL of tested extract at different concentrations or phosphate buffer for the blank or positive controls; 50 µL of DHR (5 × 10^−6^ M) and finally 50 µL of HOCl (5 × 10^−6^ M). Absorbance was read at 560 nm for 6 min at 37 °C in the microplate reader. Results were expressed as the inhibition, in IC_50_, of HOCl-induced oxidation of DHR.

#### 2.6.3. Peroxyl Radical Scavenging Activity

Peroxyl radical (ROO^●^) was generated by thermo-decomposition of AAPH at 37 °C. The ROO^●^ scavenging activity, also known as the oxygen radical absorbance capacity (ORAC) assay, was measured by the fluorescence decay of fluorescein as previously described by Ou et al. [30]. Reaction mixtures contained the following reagents dissolved in potassium phosphate buffer (75 × 10^−3^ M, pH 7.4): 150 µL of fluorescein (61.2 × 10^−9^ M); 25 µL of the tested extract at different concentrations or phosphate buffer for the blank or positive controls and 25 µL of 2,2’-Azobis(2-amidinopropane) dihydrochloride (AAPH; 19.1 × 10^−3^ M). After preparing the reaction mixture, the 96-well plate was incubated in the microplate reader during 2 h at 37 °C, where the decrease in fluorescence was measured every minute. The excitation and emission wavelengths used were 528 ± 20 nm and 485 ± 20 nm, respectively. GA and catechin were used as positive controls, while Trolox was employed as standard. Results were expressed in ratio values: slope of the sample/slope obtained for Trolox.

### 2.7. Cell Viability Assay

The 3-(4,5-dimethylthiazol-2-yl)-5-(3-carboxymethoxyphenyl)-2-(4-sulfophenyl)-2H-tetrazolium (MTT) assay was employed to access the cell viability after exposure to the extract. HFF-1 was purchased from ATCC (ATCC Number: SCRC-1041; ATCC, Manassas, VA, USA). Passages 24–26 were used. The assay was performed according to Rodrigues et al. [31]. HFF-1 was grown in DMEM medium, previously fortified with 10% of heat inactivated fetal calf serum, 1% of non-essential amino acids and 1% of antibiotic. Cells were maintained in an incubator (CO_2_ Incubator MCO-18AC, Panasonic, Osaka, Japan) at 5% CO_2_ and 37 °C and the culture medium was changed every 2 days until cells reached a good confluence. Afterwards, cells were cultured in 96-well microplates at a density of 2.5 × 10^4^ cells/mL for 24 h at 37 °C with 5% of CO_2_, in order to provide conditions for an exponential cell growth. After the period of multiplication and adherence, the medium was removed, and cells were washed with phosphate-buffer saline (PBS) solution. Following, cells were incubated with different concentrations of extract (0.1–1000 μg/mL) dissolved in the DMEM medium for 24 h at 37 °C. After the incubation period, extracts were removed and cells were washed again with PBS and subsequently MTT was added to each well. The microplate was incubated for 4 h at 37 °C with 5% of CO_2_ in the dark. After that period of incubation, DMSO was added and the microplate was put into agitation for 10 min to solubilize the MTT crystals. Positive control was made by incubating the cells only in culture medium, while negative control was made by incubating the cells only in Triton X-100. The absorbance was measured at 490 nm and at 630 nm to subtract the background. Results were expressed as percentages of cell viability.

### 2.8. Statistical Analysis

The statistical analysis was performed using IBM SPSS Statistics for Windows software (Version 24.0, IBM Corp., Armonk, NY, USA). Data was reported as mean ± standard deviation (SD) of three replications. The normal distribution and the homogeneity of variances were assessed by Shapiro–Wilk’s and Levene’s tests, respectively. For all assays, the data were normal and the homogeneity of variances confirmed. To evaluate the differences between samples, the one-way ANOVA was used. Tukey’s HSD test was employed for the post hoc comparisons of the means, being *p* < 0.05 accepted as denoting significance. To compare the same sample at different temperatures, a *t*-test was employed, being *p* < 0.05 accepted as denoting significance. GraphPad Prism 5 software (GraphPad, La Jolla, CA, USA) was employed to calculate the IC_50_ values of ROS scavenging activity.

## 3. Results and Discussion

### 3.1. Total Phenolic and Flavonoid Contents, Antioxidant and Antiradical Activities

In the present study, the influence of the extraction temperature parameter was evaluated, since previous studies have demonstrated that the temperature increase in SWE has more impact in the extraction efficiency than the solid–liquid ratio and the extraction time employed [23,24,25]. For example, Jokić et al. reported that the best result for the TPC assay was obtained when the SWE temperature was 250 °C [23]. Taking into consideration the published studies, two extreme values of temperature were employed in the present work aiming to understand their influence in the phenolic profile characterization and antioxidant activity. Table 1 summarizes the values obtained for TPC, TFC, DPPH-RSA and FRAP assays for extracts obtained by SWE performed in TN and TR vine-canes varieties, with two different extraction temperatures (125 and 250 °C).

According to Table 1, the increase of temperature from 125 to 250 °C resulted at least in 3.3-fold higher TPC values for TN and TR vine-canes varieties. Significant differences (*p* < 0.05) between the quantity of phenolic compounds extracted at 125 and 250 °C were observed. Considering the different vine-canes varieties studied, TR presented the highest TPC values (*p* < 0.05) at both tested temperatures. The highest value obtained was 181 ± 12 mg GAE/g dw for the TR extract obtained at 250 °C. These results are in line with Dorosh et al. as well as Moreira et al. that observed the same tendency for vine-canes extraction using SWE and other advanced extraction technologies: TR extracts present higher TPC values than the TN variety [12,32].

Considering the two variables studied, the values obtained for TFC were in accordance with the ones obtained for the TPC assay. As shown in Table 1, significant differences (*p* < 0.05) were observed between the TFC values obtained at 125 and 250 °C for the same vine-cane variety, as well as for the two different vine-canes varieties using the same extraction temperatures. The highest TFC was reported for TR extract obtained at 250 °C (51 ± 6 mg EE/g dw). However, the TFC values of the present study are not in agreement with the ones reported by Moreira et al. [12] since a higher TFC was reported by the authors for TN extracts obtained by SWE at 150 °C. This difference could be due to the environmental conditions observed in the harvest year, for example, as reported by other authors for other seasonal matrices also produced in the same region [33].

In what concerns to DPPH-radical scavenging assay, the capacity to reduce this radical was similar (*p* > 0.05) for both vine-canes extracts obtained at the highest temperature studied (250 °C). Nevertheless, at 125 °C, TN and TR extracts presented significant differences (*p* < 0.05). Regarding the comparison of different extraction temperatures for the same vine-cane variety, the DPPH values of the extracts obtained at 250 °C were approximately 4-fold higher than the ones obtained at 125 °C, with TR extract presenting the highest value (203 ± 22 mg TE/g dw). In fact, the increase of SWE temperature seems to be the major factor influencing the antioxidant capacity of the extracts, as previously reported by other authors [23,24,25]. The employment of high temperatures probably resulted in the formation of new compounds, which could justify the higher antioxidant activity observed [16,34]. Additionally, it was observed that the extracts with the highest TPC and TFC showed also higher DPPH^●^ scavenging capacity. The increase in antioxidant capacity caused by a higher polyphenols content was also previously reported by several other authors [11,12,35].

According to the results presented in Table 1, the obtained values for FRAP assay were in accordance to the ones obtained for DPPH-RSA assay. For instance, considering the same vine-cane variety, the results obtained for the extracts at 250 °C were almost 4-fold higher than the ones obtained at 125 °C (*p* < 0.05). Additionally, no significant differences (*p* > 0.05) were observed between the values obtained for TN and TR for the highest extraction temperature tested (250 °C). Nevertheless, significant differences (*p* < 0.05) between the two varieties were reported for extracts performed at 125 °C. The highest FRAP value was obtained for TR extract at 250 °C (202 ± 14 mg AAE/g dw), which is the same subcritical water extract that presented the highest results for the three assays previously discussed. In fact, TR variety had higher antioxidant activity than TN, which was also previously reported by Moreira et al. [12].

Based on the data previously discussed, it is possible to conclude that higher extraction temperatures resulted in higher amounts of polyphenols and flavonoids as well as higher antioxidant properties. Further, it was also demonstrated that the vine-cane variety used exerts a significant influence in the obtained results, with TR variety being a better matrix to recover bioactive compounds.

### 3.2. Identification of Phenolic Compounds by HPLC-PDA

**Figure 2:**
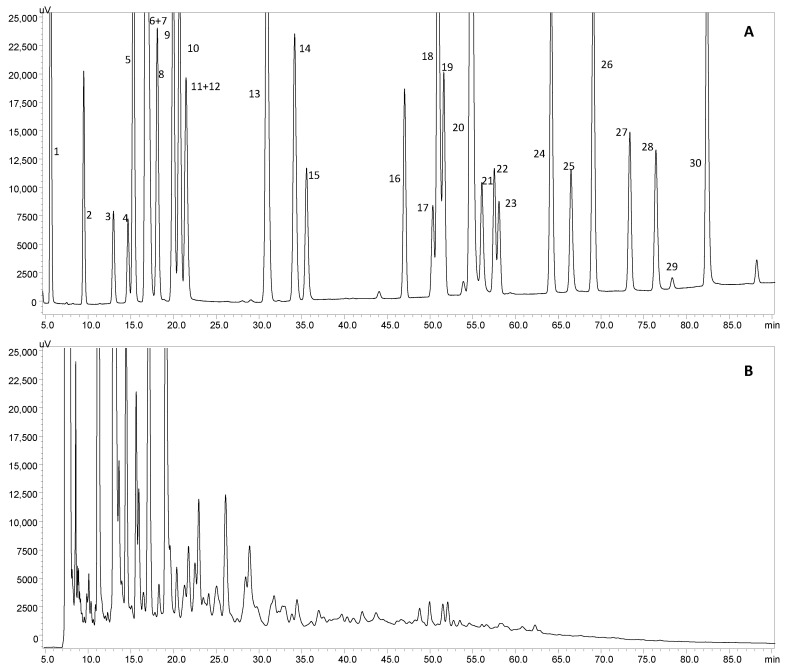
HPLC chromatograms at 280 nm for (**A**) polyphenols standard mixture and (**B**) Tinta Roriz subcritical water extract at 250 °C; peak identification: (1) gallic acid, (2) protocatechuic acid, (3) (+)-catechin, (4) 4-hydroxyphenilacetic acid, (5) 4-hydroxybenzoic acid, (6) 4-hydroxybenzaldehyde, (7) chlorogenic acid, (8) vanillic acid, (9) caffeic acid, (10) syringic acid, (11) (−)-epicatechin, (12) *p*-coumaric acid, (13) ferulic acid, (14) sinapic acid, (15) naringin, (16) rutin, (17) resveratrol, (18) quercetin-3-*O*-glucopyranoside, (19) phloridzin, (20) cinnamic acid, (21) ellagic acid, (22) myricetin, (23) kaempferol-3-*O*-glucoside, (24) kaempferol-3-*O*-rutinoside, (25) naringenin, (26) quercetin, (27) phloretin, (28) tiliroside, (29) kaempferol and (30) pinocembrin.

Due to the significant differences in the TPC, TFC, DPPH-RSA and FRAP results of the extracts obtained at 125 and 250 °C, HPLC-PDA analyses were performed to understand which phenolic compounds were the main contributors to the antioxidant properties. Figure 2 presents the HPLC chromatograms obtained for the polyphenol’s standard mixture (Figure 2A) and for TR extract obtained at 250 °C (Figure 2B). Table 2 summarizes the obtained results.

According to Table 2, phenolic acids were the major class of compounds identified and quantified, corresponding to 43% and 78% and to 38% and 80% of the total amount of phenolic compounds for TN and TR extracts obtained at 125 and 250 °C, respectively. GA was the main phenolic acid quantified, especially in the extracts obtained at 250 °C (891 ± 45 and 1066 ± 53 mg of GA/100 g dw for TN and TR respectively). In fact, GA is a compound commonly found in wines, being responsible for its characteristic astringency [14,16]. In a recent study, we reported a higher amount of phenolic acids for TN subcritical water extract obtained at 150 °C (790 ± 40 mg of phenolic acids/100 g dw) than for the CE and MAE extracts (77.3 ± 3.9 and 265 ± 13 mg of phenolic acids/100 g dw, respectively) [12]. In the same study we also observed higher amounts of phenolic compounds for TN than for TR variety contrary to the results presented in Table 2 [12].

The results discussed above suggest that the applied extraction technique significantly influences the amount of recovered bioactive compounds. Besides phenolic acids, flavanols and flavonols were the two main subclasses of flavonoid compounds present in higher amounts in the analyzed extracts. Moreover, comparing the amount of flavonols extracted by SWE at 125 °C in the present study with the amount reported in our last study at 150 °C, a higher content of flavonols was recovered when lower extraction temperature was employed, demonstrating that temperatures higher than 125 °C may cause degradation of these compounds [12]. Gabaston et al. focused on the extraction of stilbenes using SWE and obtained an amount of 362 mg of stilbenes/100 g dw, when performing the extraction at 160 °C for 5 min [22]. For the same conditions, the amount of resveratrol recovered was 130 mg/100 g dw, which is approximately 8 and 10 times more than the obtained for the extraction at 250 °C with TN and TR, respectively. Additionally, the authors pointed out that high temperatures and long periods of extraction may lead to the degradation of these compounds, which can justify the results obtained for TN and TR and presented in this paper.

Thus, the HPLC results showed that the amount of phenolic compounds quantified for the TR vine-cane variety was at least 1.31 times higher than the ones reported for TN. Regarding the two extraction temperatures tested (125 and 250 °C), the extracts obtained with the highest temperature presented higher amounts of phenolic compounds, which was already expected as the values obtained for the TPC, TFC, DPPH-RSA and FRAP assays for these extracts were also higher. In this way, the TR extract obtained at 250 °C was selected for the further assays.

### 3.3. Capacity of Scavenging Reactive Oxygen Species

Reactive oxygen species (ROS) are produced as side products of metabolic reactions. Nevertheless, in some cases these compounds are not naturally neutralized by cells, interacting with other molecules and interfering in metabolic pathways, which results in oxidative damage to cellular biomolecules [36]. Therefore, it is critical to perform assays that evaluate the scavenging capacity of ROS by extracts. Table 3 summarizes the results obtained for ROS scavenging assays.

#### 3.3.1. Superoxide Radical Scavenging Assay

As can be observed in Table 3, the TR extract presented a lower scavenging capacity of O_2_^●-^ (IC_50_ = 83.67 ± 5.84 µg/mL) than the positive controls employed (GA and catechin), which means that higher concentrations of extract would be needed to obtain the same results of the positive controls. Farhadi et al. assessed the percentage of O_2_^●-^ scavenging activity of skins, pulps, seeds, canes and leaves of five native grape cultivars in west Azerbaijan province (Iran) [37]. Even though the extracts obtained from grape skins exhibited the highest O_2_^●-^ scavenging activity (with values ranging from 86.15% to 89.92% of inhibition), vine-canes also proved to be a promising matrix (with inhibition percentages ranging from 81.46% to 86.34%). In another study performed by Barros et al., grape stem extracts were also proposed as good O_2_^●-^ scavengers [38]. The authors analyzed grape stems from seven Portuguese *V. vinifera* L. varieties, four red and three white. The IC_50_ values ranged from 970 to 2010 µg/mL for the Rabigato and Tinta Barroca grape stems varieties, respectively, being 11-fold higher than the result obtained in the present work, which demonstrates that extracts obtained from vine-canes through SWE were more efficient in scavenging O_2_^●-^.

#### 3.3.2. Hypochlorous Acid Scavenging Assay

Regarding the HOCl scavenging assay, catechin was the best scavenger presenting an IC_50_ value of 0.18 ± 0.01 μg/mL. Similarly to the O_2_^●-^ assay, the TR extract showed a lower HOCl quenching power (IC_50_ = 33.94 ± 2.95 µg/mL) than catechin and GA (IC_50_ = 1.25 ± 0.05 µg/mL). Wada et al. evaluated the scavenging capacity of hypochlorite ion (ClO^−^) by grape seed extracts using a concentration range of 0.02–2 mg/mL [39]. Two commercial grape seed extracts purchased from Mitsubishi Chemical Corporation (Kanagawa) were used in this study: extract A (proanthocyanin 99%) and B (proanthocyanin >80%). The ClO^−^ quenching capacity of grape seed extracts A and B at 1.0 mg/mL were 27.7% ± 4.2% and 22.0% ± 3.7%, respectively. The authors used five reference compounds to compare the results obtained for the extracts, namely trans-resveratrol, chalcone, cyanidin, delphinidin and pelargonidin. These grape seeds extracts exhibited lower ClO^−^ quenching effects than trans-resveratrol, cyanidin and delphinidin, but higher scavenging efficiency compared to chalcone [39].

#### 3.3.3. Peroxyl Radical Scavenging Assay

According to the obtained results (Table 3), the TR extract showed a low quenching capacity against ROO^●^ with S_sample_/S_Trolox_ lower than 1 (S_sample_/S_Trolox_ = 0.024 ± 0.001). Noteworthy, catechin presented the highest scavenging ability for ROO^●^ (S_sample_/S_Trolox_ = 7.592 ± 0.074), followed by GA (S_sample_/S_Trolox_ = 1.119 ± 0.005). In fact, the obtained subcritical water extracts were mainly composed by gallic acid and catechin, which were the main contributors to the phenolic profile of TR. However, despite their higher amount in comparison to the other phenolic compounds identified and quantified in the extracts, the levels found and recovered were lower to produce the same effect as the positive standards alone. Additionally, a synergistic effect of all the compounds present in TR extract could also negatively affect the capacity to scavenge the peroxyl radical (ROO^●^).

Although the TR extract exhibited a low capacity to quench ROO^●^, previous studies described no scavenging activity of different byproducts for this radical [40,41]. Tournour et al. also determined the ORAC values of extracts from grape pomace of four Douro’s *V. vinifera* L. varieties, including TN and TR [42]. The obtained results corresponded to 2337 ± 368 and 1054 ± 199 µmol TE/g of dw, for the TN and TR varieties, respectively. According to the authors, TN presented the best ORAC values, being therefore also better than the ones obtained for TR. Nevertheless, the highest TPC and the best result for the DPPH-RSA assay, obtained in the study aforementioned, also corresponded to the TN variety, which suggest that the radical scavenging capacity is directly related to the results obtained by both assays. In the present study, the TR variety was the one that achieved the highest values for the TPC and DPPH assays, which supports the choice made to use this extract for further studies. Barros et al. also assessed the ORAC values of seven Portuguese grape stems varieties extracts, obtained through sonication baths [38]. The results obtained ranged from 40.26 to 150.79 mM Trolox/100 g of dried grape stems. A direct comparison with the values from the present work cannot be made, but the results reported by these authors supported the ones obtained in O_2_^●-^ scavenging assay, where higher values were reported for the red vine varieties.

Among the ROS studied, the highest scavenging efficiencies of the TR extract were observed for HOCl (IC_50_ = 33.94 ± 2.95 µg/mL) and O_2_^●-^ (IC_50_ = 83.67 ± 5.84 µg/mL). According to the results observed for these radical scavenging assays, TR vine-canes extracts obtained by SWE at 250 °C showed promising results for applications in health-related products.

### 3.4. Cell Viability Studies

**Figure 3:**
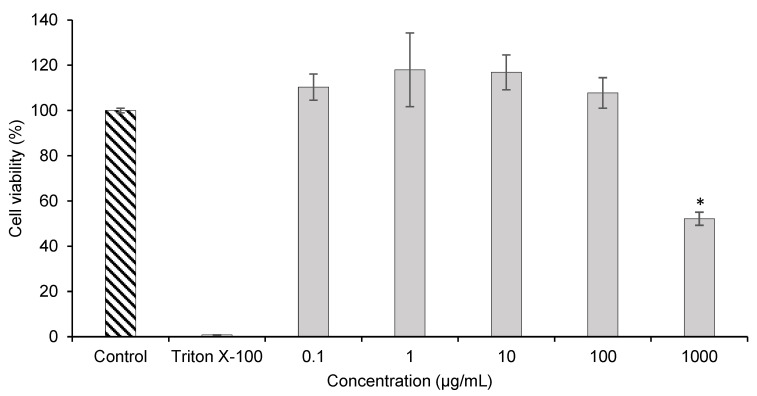
Effect of different concentrations of TR extract on the metabolic activity of HFF-1 cells, measured by the MTT assay. Values were expressed as mean ± SD (*n*=4). * means significant differences (*p* < 0.05).

The cell viability results are represented in Figure 3 and were obtained after exposure of HFF-1 to different concentrations of TR extract (0.1; 1.0; 10; 100 and 1000 µg/mL).

As it is possible to observe, extract concentrations under 100 µg/mL did not result in a reduction of HFF-1 cellular viability. For these concentrations, the cell viability was higher than 100%. However, at a concentration of 1000 µg/mL there was a considerable reduction of cell viability to 52.15% (*p* < 0.05). Manca et al. incorporated grape seed and vine-canes extracts in vesicular systems designed for topical applications and analyzed their cell viability in HFF-1 [43]. In the MTT test, the authors incubated the cells solely with the extracts to serve as a comparison value and with the vesicles in four different concentrations, namely 0.2, 2, 20 and 40 µg/mL, for 48 h. After the incubation time, the best values were obtained for the cells incubated only with the grape seed and vine-canes extracts (>100%). These results are in accordance with the ones obtained for the TR extract, since until the concentration of 100 µg/mL the cell viability was also above 100%.

## 4. Conclusions

The present work proposes an alternative application for the reuse of vine-canes, an agro-industrial byproduct derived from grape production. Grapes are one of the major fruit crops produced throughout the world and after harvesting season the vines need to be pruned, which generates every year large amounts of wastes. The challenge is to find alternative solutions to the presently used applications (incorporation in the soil), considering not only economic profits but, mostly, environmental impacts. The results obtained proved that SWE is a suitable environmentally friendly technique for vine-canes extraction of bioactive compounds with antioxidant properties. The TR extract obtained the highest results with the highest temperature tested (250 °C), achieving a TPC of 181 ± 12 mg GAE/g dw and an antioxidant activity through the FRAP assay of 202 ± 14 mg AAE/g dw. In general, a tendency was observed: extracts with higher phenolic content also presented higher flavonoid content, higher DPPH-RSA and FRAP values. Indeed, extracts obtained at 250 °C presented higher results than the ones obtained at 125 °C.

Regarding the HPLC-PDA analysis, the TR variety presented the higher amount of individual phenolic compounds for the highest extraction temperature tested. The phenolic compound present in higher amounts was GA (1145 ± 57 and 1502 ± 75 mg of GA/100 g of dw for the TN and TR varieties extracts obtained at 250 °C, respectively), which seems to be the main compound contributing for the high antioxidant activity of these extracts. Taking into consideration the results obtained, TR extract prepared at 250 °C was selected for the radicals scavenging and cell assays, revealing a good scavenging capacity of oxygen species as well as no adverse effects on HFF-1 were detected until a concentration of 100 µg/mL.

Considering the portion that grape production occupies in cultivation of food crops worldwide, the use of vine-canes in added value products could have a significant impact in the sustainability of food and beverage industries. Indeed, final applications in functional foods or cosmetics would be of extreme interest. As future perspectives, it would be interesting to test a scale up for SWE, since at the industrial level it might be more profitable.

## Figures and Tables

**Table 1:** Total phenolic content (TPC, results expressed in mg gallic acid equivalents (GAE)/g dw), total flavonoid content (TFC, results expressed in mg epicatechin equivalents (EE)/g dw), 2,2-diphenyl-1-picrylhydrazyl radical scavenging activity (DPPH-RSA, results expressed in milligram Trolox equivalents (TE)/g dw) and ferric reduction antioxidant power (FRAP, results expressed in milligram ascorbic acid equivalents (AAE)/g dw) of subcritical water extracts from Touriga Nacional (TN) and Tinta Roriz (TR) vine-canes varieties. Results were expressed as mean ± standard deviation.

Temp. (°C)	TPC (mg GAE/g dw)		TFC (mg EE/g dw)		DPPH-RSA (mg TE/g dw)		FRAP (mg AAE/g dw)	
	TN	TR	TN	TR	TN	TR	TN	TR
125	47 ± 7	55 ± 2 ^#^	17 ± 2	20 ± 1 ^#^	51 ± 9	56 ± 2 ^#^	46 ± 7	53 ± 2 ^#^
250	165 ± 8 ^*^	181 ± 12 ^*,#^	46 ± 3 ^*^	51 ± 6^*,#^	202 ± 22^*^	203 ± 22^*^	186 ± 21 ^*^	202 ± 14 ^*^

For each assay (TPC, TFC, DPPH-RSA and FRAP), ^#^ for the same extraction temperature means that the vine-cane variety produces statistically significant differences (*p* < 0.05); * for the same vine-cane variety means that different extraction temperatures produce statistically significant differences (*p* < 0.05).

**Table 2:** Content of the identified phenolic compounds in Touriga Nacional (TN) and Tinta Roriz (TR) extracts obtained through subcritical water extraction (SWE) at 125 and 250 °C. Results were expressed as mean ± standard deviations (milligrams of compound/100g dw, *n*=3).

Compound	TN		TR	
	125 °C	250 °C	125 °C	250 °C
Phenolic acids				
Gallic acid	60.1 ± 3.0	891 ± 45	74.2 ± 3.7	1066 ± 53
Protocatechuic acid	33.8 ± 1.7	14.5 ± 0.7	33.4 ± 1.7	21.2 ± 1.1
4-hydroxyphenilacetic acid	16.8 ± 0.8	62.6 ± 3.1	49.2 ± 2.5	134 ± 7
4-hydroxybenzoic acid	9.2 ± 0.5	22.6 ± 1.1	8.4 ± 0.4	44.9 ± 2.2
4-hydroxybenzaldehyde	4.9 ± 0.2	7.5 ± 0.4	4.7 ± 0.2	9.8 ± 0.5
Chlorogenic acid	6.2 ± 0.3	23.2 ± 1.2	7.4 ± 0.4	44.3 ± 2.2
Vanillic acid	15.0 ± 0.7	15.6 ± 0.8	13.5 ± 0.7	31.6 ± 1.6
Caffeic acid	14.9 ± 0.7	13.6 ± 0.7	14.4 ± 0.7	19.6 ± 1.0
Syringic acid	ND ^a^	37.9 ± 1.9	<LOD ^b^	65.6 ± 3.3
*p*-coumaric acid	17.2 ± 0.9	16.0 ± 0.8	22.9 ± 1.1	21.7 ± 1.1
Ferulic acid	21.9 ± 1.1	18.9 ± 0.9	24.5 ± 1.2	19.6 ± 1.0
Sinapic acid	17.1 ± 0.9	14.4 ± 0.7	22.0 ± 1.0	12.2 ± 0.6
Cinnamic acid	11.1 ± 0.6	8.1 ± 0.4	12.2 ± 0.6	11.1 ± 0.5
∑Phenolic acids	228 ± 11	1145 ± 57	286 ± 14	1502 ± 75
Flavanols				
(+)-Catechin	102 ± 5	181 ± 9	245 ± 12	216 ± 11
(-)-Epicatechin	3.9 ± 0.2	3.1 ± 0.2	17.2 ± 0.9	14.6 ± 0.7
∑Flavanols	106 ± 5	184 ± 9	262 ± 13	231 ± 12
Flavanones				
Naringin	<LOD	8.6 ± 0.4	<LOD	14.7 ± 0.7
Naringenin	4.5 ± 0.2	2.4 ± 0.1	6.1 ± 0.3	3.3 ± 0.2
∑Flavanones	4.5 ± 0.2	11.0 ± 0.6	6.1 ± 0.3	18.0 ± 0.9
Flavonols				
Rutin	3.1 ± 0.2	9.2 ± 0.5	1.4 ± 0.1	15.4 ± 0.8
Quercetin-3-*O*-glucopyranoside	4.4 ± 0.2	5.2 ± 0.3	4.1 ± 0.2	10.7 ± 0.5
Myricetin	84.3 ± 4.2	14.6 ± 0.7	86.4 ± 4.3	16.2 ± 0.8
Kaempferol-3-*O*-glucoside	9.4 ± 0.5	ND	10.1 ± 0.5	8.6 ± 0.4
Kaempferol-3-*O*-rutinoside	4.3 ± 0.2	2.3 ± 0.1	4.7 ± 0.2	7.0 ± 0.4
Quercetin	40.9 ± 2.0	24.9 ± 1.2	40.7 ± 2.0	30.8 ± 1.5
Kaempferol	34.6 ± 1.7	23.2 ± 1.2	41.6 ± 2.1	24.2 ± 1.2
∑Flavonols	181 ± 9	79.4 ± 4.0	189 ± 9	113 ± 6
Stilbenes				
Resveratrol	8.0 ± 0.4	15.8 ± 0.8	10.5 ± 0.5	13.1 ± 0.7
∑Stilbenes	8.0 ± 0.4	15.8 ± 0.8	10.5 ± 0.5	13.1 ± 0.7
Others				
Phloridzin	<LOD	3.7 ± 0.2	<LOD	8.0 ± 0.4
Phloretin	ND	<LOD	<LOD	<LOD
∑Others	0.0 ± 0.0	3.7 ± 0.2	0.0 ± 0.0	8.0 ± 0.4
∑All phenolic compounds	527	1440	755	1884

^a^ ND: not detected; ^b^ LOD: limit of detection.

**Table 3:** Superoxide (O_2_^●-^), hypochlorous acid (HOCl) and peroxyl radical (ROO^●^) scavenging activities of Tinta Roriz (TR) subcritical water extract obtained at 250 °C.

Reactive Species	O_2_^●-^	HOCl	ROO^●^
	IC_50_ (µg/mL)		S_sample_/S_Trolox_^a^
**TR sample**	83.67 ± 5.84	33.94 ± 2.95	0.024 ± 0.001
**Gallic acid**	5.18 ± 0.19	1.25 ± 0.05	1.119 ± 0.005
**Catechin**	48.99 ± 0.75	0.18 ± 0.01	7.592 ± 0.074

IC_50_ = in-vitro inhibitory concentration, expressed in µg/mL, required to scavenge 50% of the generated reactive oxygen species (mean ± SD, *n* = 3). ^a^ Results for ROO^●^ scavenging activity are expressed as slop ratio between samples or positive controls and Trolox. S_sample_ = slope of extract/positive controls curves and S_Trolox_ = slope of Trolox curve.

## References

[1] Kuzmina K., Prendeville S., Walker D., Charnley F. (2019). Future scenarios for fast-moving consumer goods in a circular economy. Futures.

[2] Roser M., Ritchie H., Ortiz-Ospina E. World Population Growth. https://ourworldindata.org/world-population-growth.

[3] Siracusa L., Ruberto G., Watson R. (2018). Not only what is food is good-polyphenols from edible and nonedible vegetables waste. Polyphenols in Plants.

[4] Clark G., Aghion P., Steven D. (2005). The industrial revolution. Handbook of Economic Growth.

[5] Russ W., Schnappinger M., Oreopoulou V., Russ W. (2007). Waste related to the food industry: A challenge in material loops. Utilization of By-Products and Treatment of Waste in the Food Industry.

[6] Oliveira L.S., Franca A.S., Greco L., Bruno M. (2008). Low-cost adsorbents from agri-food wastes. Food Sciences Technology: New Research.

[7] Rodríguez R., Jiménez A., Fernández-Bolaños J., Guillén R., Heredia A. (2006). Dietary fibre from vegetable products as source of functional ingredients. Trends Food Sci. Technol..

[8] Devesa-Rey R., Vecino X., Varela-Alende J.L., Barral M.T., Cruz J.M., Moldes A.B. (2011). Valorization of winery waste vs. The costs of not recycling. Waste Managem..

[9] OIV—International Organisation of Vine and Wine (OIV) (2016). OIV Statistical Report on World Vitiviniculture.

[10] Rajha H., Darra N., Hobaika Z., Boussetta N., Vorobiev E., Maroun R., Louka N. (2014). Extraction of total phenolic compounds, flavonoids, anthocyanins and tannins from grape byproducts by response surface methodology. Influence of solid-liquid ratio, particle size, time, temperature and solvent mixtures on the optimization process. Food Nutr. Sci..

[11] Gullón B., Eibes G., Moreira M.T., Dávila I., Labidi J., Gullón P. (2017). Antioxidant and antimicrobial activities of extracts obtained from the refining of autohydrolysis liquors of vine shoots. Ind. Crops Prod..

[12] Moreira M.M., Barroso M.F., Porto J.V., Ramalhosa M.J., Švarc-Gajić J., Estevinho L., Morais S., Delerue-Matos C. (2018). Potential of portuguese vine shoot wastes as natural resources of bioactive compounds. Sci. Total Environ..

[13] Rajha H.N., Boussetta N., Louka N., Maroun R.G., Vorobiev E. (2014). A comparative study of physical pretreatments for the extraction of polyphenols and proteins from vine shoots. Food Res. Int..

[14] Sanchez-Gomez R., Zalacain A., Alonso G.L., Salinas M.R. (2014). Vine-shoot waste aqueous extracts for re-use in agriculture obtained by different extraction techniques: Phenolic, volatile, and mineral compounds. J. Agr. Food Chem..

[15] Karacabey E., Mazza G., Bayındırlı L., Artık N. (2012). Extraction of bioactive compounds from milled grape canes (*Vitis vinifera*) using a pressurized low-polarity water extractor. Food Bioprocess Tech..

[16] Delgado-Torre M.P., Ferreiro-Vera C., Priego-Capote F., Perez-Juan P.M., Luque de Castro M.D. (2012). Comparison of accelerated methods for the extraction of phenolic compounds from different vine-shoot cultivars. J. Agr. Food Chem..

[17] Jiménez Gómez S., Cartagena Causapé M.C., Arce Martínez A. (1993). Distribution of nutrients in anaerobic digestion of vine shoots. Bioresour. Technol..

[18] Tag A.T., Duman G., Ucar S., Yanik J. (2016). Effects of feedstock type and pyrolysis temperature on potential applications of biochar. J. Anal. Appl. Pyrol..

[19] Dávila I., Gullón B., Labidi J., Gullón P. (2019). Multiproduct biorefinery from vine shoots: Bio-ethanol and lignin production. Renew. Energy.

[20] Fernandes M.J., Moreira M.M., Paíga P., Dias D., Bernardo M., Carvalho M., Lapa N., Fonseca I., Morais S., Figueiredo S. (2019). Evaluation of the adsorption potential of biochars prepared from forest and agri-food wastes for the removal of fluoxetine. Bioresour. Technol..

[21] Jiménez L., Angulo V., Ramos E., De la Torre M.J., Ferrer J.L. (2006). Comparison of various pulping processes for producing pulp from vine shoots. Ind. Crops Prod..

[22] Gabaston J., Leborgne C., Valls J., Renouf E., Richard T., Waffo-Teguo P., Mérillon J.-M. (2018). Subcritical water extraction of stilbenes from grapevine by-products: A new green chemistry approach. Ind. Crops. Prod..

[23] Jokić S., Gagić T., Knez Ž., Banožić M., Škerget M. (2019). Separation of active compounds from tobacco waste using subcritical water extraction. J. Supercrit. Fluids.

[24] Nile S.H., Nile A., Gansukh E., Baskar V., Kai G. (2019). Subcritical water extraction of withanosides and withanolides from ashwagandha (*Withania somnifera* l) and their biological activities. Food Chem. Toxicol..

[25] Lee J.-H., Ko M.-J., Chung M.-S. (2018). Subcritical water extraction of bioactive components from red ginseng (*Panax ginseng* c.A. Meyer). J. Supercrit. Fluids.

[26] Singleton V.L., Rossi J.A.J. (1965). Colorimetry of total phenolics with phosphomolybdic–phosphotungstic acid reagents. Am. J. Enol. Viticult..

[27] Paz M., Gúllon P., Barroso M.F., Carvalho A.P., Domingues V.F., Gomes A.M., Becker H., Longhinotti E., Delerue-Matos C. (2015). Brazilian fruit pulps as functional foods and additives: Evaluation of bioactive compounds. Food Chem..

[28] Benzie I.F.F., Strain J.J. (1996). The ferric reducing ability of plasma (frap) as a measure of “antioxidant power”: The FRAP assay. Anal. Biochem..

[29] Pistón M., Machado I., Branco C.S., Cesio V., Heinzen H., Ribeiro D., Fernandes E., Chisté R.C., Freitas M. (2014). Infusion, decoction and hydroalcoholic extracts of leaves from artichoke (*Cynara cardunculus* l. Subsp. Cardunculus) are effective scavengers of physiologically relevant ROS and RNS. Food Res. Int..

[30] Ou B., Hampsch-Woodill M., Prior R.L. (2001). Development and validation of an improved oxygen radical absorbance capacity assay using fluorescein as the fluorescent probe. J. Agr. Food Chem..

[31] Rodrigues F., Palmeira-de-Oliveira A., das Neves J., Sarmento B., Amaral M.H., Oliveira M.B. (2013). *Medicago* spp. extracts as promising ingredients for skin care products. Ind. Crops. Prod..

[32] Dorosh O., Moreira M.M., Rodrigues F., Peixoto A.F., Freire C., Morais S., Delerue-Matos C. (2020). Vine-canes valorisation: Ultrasound-assisted extraction from lab to pilot scale. Molecules.

[33] Rodrigues F., Santos J., Pimentel F.B., Braga N., Palmeira-de-Oliveira A., Oliveira M.B.P.P. (2015). Promising new applications of *Castanea sativa* shell: Nutritional composition, antioxidant activity, amino acids and vitamin e profile. Food Funct..

[34] Plaza M., Amigo-Benavent M., del Castillo M.D., Ibáñez E., Herrero M. (2010). Neoformation of antioxidants in glycation model systems treated under subcritical water extraction conditions. Food Res. Int..

[35] Anastasiadi M., Pratsinis H., Kletsas D., Skaltsounis A.-L., Haroutounian S.A. (2012). Grape stem extracts: Polyphenolic content and assessment of their *in vitro* antioxidant properties. LWT Food Sci. Technol..

[36] Nunes M.A., Rodrigues F., Oliveira M.B.P.P., Galanakis C. (2017). Chapter 11—Grape processing by-products as active ingredients for cosmetic proposes. Handbook of Grape Processing by-Products.

[37] Farhadi K., Esmaeilzadeh F., Hatami M., Forough M., Molaie R. (2016). Determination of phenolic compounds content and antioxidant activity in skin, pulp, seed, cane and leaf of five native grape cultivars in west azerbaijan province, iran. Food Chem..

[38] Barros A., Gironés-Vilaplana A., Teixeira A., Collado-González J., Moreno D.A., Gil-Izquierdo A., Rosa E., Domínguez-Perles R. (2014). Evaluation of grape (*Vitis vinifera* L.) stems from portuguese varieties as a resource of (poly)phenolic compounds: A comparative study. Food Res. Int..

[39] Wada M., Kido H., Ohyama K., Ichibangase T., Kishikawa N., Ohba Y., Nakashima M.N., Kuroda N., Nakashima K. (2007). Chemiluminescent screening of quenching effects of natural colorants against reactive oxygen species: Evaluation of grape seed, monascus, gardenia and red radish extracts as multi-functional food additives. Food Chem..

[40] Almeida D., Pinto D., Santos J., Vinha A.F., Palmeira J., Ferreira H.N., Rodrigues F., Oliveira M.B.P.P. (2018). Hardy kiwifruit leaves (*Actinidia arguta*): An extraordinary source of value-added compounds for food industry. Food Chem..

[41] Pinto D., Diaz Franco S., Silva A.M., Cupara S., Koskovac M., Kojicic K., Soares S., Rodrigues F., Sut S., Dall’Acqua S. (2019). Chemical characterization and bioactive properties of a coffee-like beverage prepared from *Quercus cerris* kernels. Food Funct..

[42] Tournour H.H., Segundo M.A., Magalhães L.M., Barreiros L., Queiroz J., Cunha L.M. (2015). Valorization of grape pomace: Extraction of bioactive phenolics with antioxidant properties. Ind. Crops Prod..

[43] Manca M.L., Firoznezhad M., Caddeo C., Marongiu F., Escribano-Ferrer E., Sarais G., Peris J.E., Usach I., Zaru M., Manconi M. (2019). Phytocomplexes extracted from grape seeds and stalks delivered in phospholipid vesicles tailored for the treatment of skin damages. Ind. Crops. Prod..

